# Supplementary material for: Clinical characteristics and a diagnostic model for high-altitude pulmonary edema in habitual low altitude dwellers
Source: PeerJ. 2024 Sep 26;12:e18084. doi: 10.7717/peerj.18084 (PMC11439376; doi:10.7717/peerj.18084)
Supplement: Supplemental Information 1 [file peerj-12-18084-s001.doc]

**Table 1 Descriptive summary of study sample statistics by groups**

| **Study Variable** | **HAPE & non-HAPE** | | **P value** |
| --- | --- | --- | --- |
|  | **HAPE group (N = 604)** | **Control group (N = 651)** |  |
| **gender (n/%)** |  |  | < 0.01 |
| **male** | 459 (76.37) | 383 (58.83) |  |
| **female** | 145 (23.63) | 268 (41.17) |  |
| **Age(y) (n/%)** |  |  | 0.37 |
| **18-34** | 221 (36.59) | 269 (41.32) |  |
| **35-49** | 263 (43.54) | 259 (39.78) |  |
| **50-64** | 108 (17.88) | 112 (17.20) |  |
| **65-** | 12 (1.99) | 11 (1.70) |  |
| **BMI(Kg/m^2^) (n/%)** |  |  | < 0.01 |
| **<18.5** | 11 (1.82) | 51 (7.83) |  |
| **18.5-24.9** | 304 (50.33) | 424 (65.13) |  |
| **25-** | 289 (47.85) | 176 (27.04) |  |
| **HR (per min)** |  |  | < 0.01 |
| **Median** | 105.00 | 83.00 |  |
| **IQR (25-75)** | 90.00, 118.00 | 74.00, 92.00 |  |
| **BR (per min)** |  |  | < 0.01 |
| **Median** | 23.00 | 20.00 |  |
| **IQR (25-75)** | 22.00, 25.00 | 20.00, 21.00 |  |
| **SP (mmHg)** |  |  | < 0.01 |
| **Median** | 122.00 | 120.00 |  |
| **IQR (25-75)** | 110.00, 138.00 | 102.00, 130.00 |  |
| **DP (mmHg)** |  |  | < 0.01 |
| **Median** | 80.00 | 78.00 |  |
| **IQR (25-75)** | 72.00, 90.00 | 66.00, 90.00 |  |
| **MAP (mmHg)** |  |  | <0.01 |
| **Median** | 94.00 | 91.00 |  |
| **IQR (25-75)** | 86.70, 106.00 | 78.70, 103.30 |  |
| **WBC (10^9^/L)** |  |  | <0.01 |
| **Median** | 10.60 | 8.20 |  |
| **IQR (25-75)** | 7.90, 13.80 | 6.40, 10.80 |  |
| **LYMPH% (%)** |  |  | <0.01 |
| **Median** | 17.90 | 23.10 |  |
| **IQR (25-75)** | 13.10, 23.50 | 16.60, 30.20 |  |
| **NEUT% (%)** |  |  | <0.01 |
| **Median** | 74.90 | 69.30 |  |
| **IQR (25-75)** | 68.50, 80.40 | 62.20, 76.80 |  |
| **HGB (g/L)** |  |  | <0.01 |
| **Median** | 159.00 | 166.00 |  |
| **IQR (25-75)** | 143.00, 174.00 | 149.00, 183.00 |  |
| **RBC (10^12^/L)** |  |  | <0.01 |
| **Median** | 5.00 | 5.20 |  |
| **IQR (25-75)** | 4.60, 5.50 | 4.70, 5.60 |  |
| **HCT (%)** |  |  | <0.01 |
| **Median** | 46.90 | 49.50 |  |
| **IQR (25-75)** | 42.30, 51.00 | 44.80, 54.50 |  |
| **MCV (fL)** |  |  | <0.01 |
| **Median** | 93.40 | 96.60 |  |
| **IQR (25-75)** | 90.00, 97.30 | 93.10, 99.60 |  |
| **MCH (pg)** |  |  | <0.01 |
| **Median** | 32.00 | 32.30 |  |
| **IQR (25-75)** | 30.50, 33.30 | 31.00, 33.60 |  |
| **MCHC (g/L)** |  |  | <0.01 |
| **Median** | 337.00 | 334.00 |  |
| **IQR (25-75)** | 326.00, 353.00 | 325.00, 342.00 |  |
| **PLT (10^9^/L)** |  |  | <0.01 |
| **Median** | 210.00 | 227.00 |  |
| **IQR (25-75)** | 168.00, 250.00 | 192.00, 275.00 |  |
| **MPV (fL)** |  |  | <0.01 |
| **Median** | 8.50 | 8.30 |  |
| **IQR (25-75)** | 7.80, 9.30 | 7.70, 8.90 |  |
| **PCT (%)** |  |  | <0.01 |
| **Median** | 0.20 | 0.20 |  |
| **IQR (25-75)** | 0.10, 0.20 | 0.20, 0.20 |  |
| **PLCR (%)** |  |  | <0.01 |
| **Median** | 26.50 | 25.00 |  |
| **IQR (25-75)** | 21.20, 33.00 | 20.50, 30.70 |  |
| **Cl (mmol/L)** |  |  | 0.95 |
| **Median** | 101.00 | 100.80 |  |
| **IQR (25-75)** | 98.40, 103.90 | 98.40, 104.00 |  |
| **tCa (mmol/L)** |  |  | <0.01 |
| **Median** | 2.20 | 2.40 |  |
| **IQR (25-75)** | 2.10, 2.40 | 2.30, 2.50 |  |
| **AST (U/L)** |  |  | <0.01 |
| **Median** | 21.00 | 19.00 |  |
| **IQR (25-75)** | 17.00, 28.00 | 16.00, 25.00 |  |
| **AST/ALT** |  |  | <0.01 |
| **Median** | 0.80 | 0.90 |  |
| **IQR (25-75)** | 0.60, 1.10 | 0.70, 1.30 |  |
| **TP (g/L)** |  |  | <0.01 |
| **Median** | 70.20 | 72.40 |  |
| **IQR (25-75)** | 64.90, 76.10 | 67.10, 77.60 |  |
| **ALB (g/L)** |  |  | <0.01 |
| **Median** | 43.80 | 45.50 |  |
| **IQR (25-75)** | 40.00, 47.00 | 42.40, 48.80 |  |
| **GLO (g/L)** |  |  | 0.2 |
| **Median** | 26.50 | 27.00 |  |
| **IQR (25-75)** | 22.70, 30.00 | 23.00, 30.30 |  |
| **A/G** |  |  | <0.01 |
| **Median** | 1.60 | 1.70 |  |
| **IQR (25-75)** | 1.50, 1.90 | 1.50, 1.90 |  |
| **TBIL (umol/L)** |  |  | 0.59 |
| **Median** | 17.10 | 16.40 |  |
| **IQR (25-75)** | 12.30, 23.30 | 11.60, 24.50 |  |
| **DBIL (umol/L)** |  |  | 0.69 |
| **Median** | 4.50 | 4.40 |  |
| **IQR (25-75)** | 3.00, 6.80 | 3.00, 6.80 |  |
| **IBIL (umol/L)** |  |  | <0.01 |
| **Median** | 4.00 | 11.80 |  |
| **IQR (25-75)** | 1.90, 8.40 | 8.10, 17.10 |  |
| **LDH (U/L)** |  |  | <0.01 |
| **Median** | 192.00 | 172.00 |  |
| **IQR (25-75)** | 162.00, 234.00 | 146.00, 205.00 |  |
| **UREA (mmol/L)** |  |  | <0.01 |
| **Median** | 5.20 | 4.50 |  |
| **IQR (25-75)** | 4.10, 6.40 | 3.70, 5.70 |  |

* Continuous variables with a nonnormal distribution were reported as the median with interquartile range (IQR) expressed as the twenty‐fifth to the seventy‐fifth percentile. Continuous variables were compared using Mann-Whitney U test and categorical variables were compared using Wilcoxon’s rank test.

**Table 2 Summary of study variables stratified by data set**

| **Study Variable** | **Data set** | | **P value** |
| --- | --- | --- | --- |
|  | **Training (N=864)** | **Validation (N=391)** |  |
| **Gender (n/%)** |  |  | 0.8 |
| **male** | 582 (67.36) | 260 (66.50) |  |
| **female** | 282 (32.64) | 131 (33.50) |  |
| **Age(y) (n/%)** |  |  | 0.96 |
| **18-34** | 341 (39.5%) | 149 (38.1%) |  |
| **35-49** | 358 (41.4%) | 164 (41.9%) |  |
| **50-64** | 149 (17.2%) | 71 (18.2%) |  |
| **65-** | 16 (1.9%) | 7 (1.8%) |  |
| **BMI(Kg/m2) (n/%)** |  |  | 0.99 |
| **<18.5** | 43 (4.98) | 19 (4.86) |  |
| **18.5-24.9** | 502 (58.10) | 226 (57.80) |  |
| **25-** | 319 (36.92) | 146 (37.34) |  |
| **HR (per min)** |  |  | 0.46 |
| **Median** | 90 | 94 |  |
| **IQR (25-75)** | 80.00, 108.00 | 78.00, 110.00 |  |
| **BR (per min)** |  |  | 0.9 |
| **Median** | 22 | 21 |  |
| **IQR (25-75)** | 20.00, 24.00 | 20.00, 24.00 |  |
| **SP (mmHg)** |  |  | 0.73 |
| **Median** | 120 | 120 |  |
| **IQR (25-75)** | 108.00, 136.00 | 108.00, 136.00 |  |
| **DP (mmHg)** |  |  | 0.99 |
| **Median** | 80 | 80 |  |
| **IQR (25-75)** | 70.00, 90.00 | 70.00, 90.00 |  |
| **MAP (mmHg)** |  |  | 0.95 |
| **Median** | 93.3 | 92 |  |
| **IQR (25-75)** | 83.30, 104.00 | 83.30, 105.30 |  |
| **WBC (109/L)** |  |  | 0.3 |
| **Median** | 9.1 | 9.4 |  |
| **IQR (25-75)** | 7.00, 12.00 | 7.00, 12.80 |  |
| **LYMPH% (%)** |  |  | 0.27 |
| **Median** | 20.7 | 19.9 |  |
| **IQR (25-75)** | 15.10, 27.50 | 14.00, 27.30 |  |
| **NEUT% (%)** |  |  | 0.1 |
| **Median** | 71.9 | 72.6 |  |
| **IQR (25-75)** | 64.90, 78.10 | 64.70, 79.80 |  |
| **HGB (g/L)** |  |  | 0.97 |
| **Median** | 162 | 163 |  |
| **IQR (25-75)** | 146.00, 180.00 | 146.00, 179.00 |  |
| **RBC (1012/L)** |  |  | 0.5 |
| **Median** | 5.1 | 5.2 |  |
| **IQR (25-75)** | 4.60, 5.60 | 4.70, 5.60 |  |
| **HCT (%)** |  |  | 0.87 |
| **Median** | 48.2 | 48.1 |  |
| **IQR (25-75)** | 43.40, 53.00 | 43.40, 53.00 |  |
| **MCV (fL)** |  |  | 0.32 |
| **Median** | 95.4 | 94.6 |  |
| **IQR (25-75)** | 91.60, 98.90 | 91.30, 98.80 |  |
| **MCH (pg)** |  |  | 0.66 |
| **Median** | 32.2 | 32.1 |  |
| **IQR (25-75)** | 30.80, 33.50 | 30.60, 33.40 |  |
| **MCHC (g/L)** |  |  | 0.63 |
| **Median** | 335 | 335 |  |
| **IQR (25-75)** | 326.00, 346.00 | 326.00, 346.00 |  |
| **PLT (109/L)** |  |  | 0.13 |
| **Median** | 222 | 215 |  |
| **IQR (25-75)** | 180.50, 261.50 | 176.00, 259.00 |  |
| **MPV (fL)** |  |  | 0.17 |
| **Median** | 8.3 | 8.4 |  |
| **IQR (25-75)** | 7.70, 9.10 | 7.90, 9.20 |  |
| **PCT (%)** |  |  | 0.35 |
| **Median** | 0.2 | 0.2 |  |
| **IQR (25-75)** | 0.20, 0.20 | 0.20, 0.20 |  |
| **PLCR (%)** |  |  | 0.06 |
| **Median** | 25.3 | 26.1 |  |
| **IQR (25-75)** | 20.40, 31.60 | 21.90, 32.40 |  |
| **tCa (mmol/L)** |  |  | 0.98 |
| **Median** | 2.3 | 2.3 |  |
| **IQR (25-75)** | 2.20, 2.40 | 2.20, 2.40 |  |
| **ALT (U/L)** |  |  | 0.61 |
| **Median** | 23 | 23 |  |
| **IQR (25-75)** | 15.00, 36.00 | 16.00, 36.00 |  |
| **AST (U/L)** |  |  | 0.34 |
| **Median** | 20 | 20 |  |
| **IQR (25-75)** | 16.00, 27.00 | 16.00, 26.00 |  |
| **AST/ALT** |  |  | 0.07 |
| **Median** | 0.9 | 0.8 |  |
| **IQR (25-75)** | 0.70, 1.20 | 0.60, 1.20 |  |
| **TP (g/L)** |  |  | 0.97 |
| **Median** | 71.4 | 71.4 |  |
| **IQR (25-75)** | 65.80, 76.70 | 66.30, 77.30 |  |
| **ALB (g/L)** |  |  | 0.93 |
| **Median** | 44.7 | 45 |  |
| **IQR (25-75)** | 41.40, 48.00 | 41.00, 48.30 |  |
| **A/G** |  |  | 0.43 |
| **Median** | 1.7 | 1.7 |  |
| **IQR (25-75)** | 1.50, 1.90 | 1.50, 1.90 |  |
| **IBIL (umol/L)** |  |  | 0.42 |
| **Median** | 8.4 | 8.4 |  |
| **IQR (25-75)** | 4.00, 13.80 | 3.30, 13.60 |  |
| **LDH (U/L)** |  |  | 0.98 |
| **Median** | 182 | 179.3 |  |
| **IQR (25-75)** | 152.50, 219.00 | 154.00, 218.00 |  |
| **UREA (mmol/L)** |  |  | 0.16 |
| **Median** | 4.7 | 4.9 |  |
| **IQR (25-75)** | 3.80, 6.00 | 3.90, 6.20 |  |

* Continuous variables with a nonnormal distribution were reported as the median with interquartile range (IQR) expressed as the twenty‐fifth to the seventy‐fifth percentile. Continuous variables were compared using Mann-Whitney U test and categorical variables were compared using Wilcoxon’s rank test.

**Table 3 Predictors for HAPE in final regression model for training dataset**

| **Intercept and** | **β Coefficient** | **OR (95% CI)** | **P Value** |
| --- | --- | --- | --- |
| **Variable** |
| **Intercept** | 5.635 | NA | 0.009 |
| **gender(1)** | -1.015 | 0.362(0.240,0.547) | 0.000 |
| **age** |  |  | 0.318 |
| age(1) | -0.067 | 0.935(0.653,1.34) | 0.715 |
| age(2) | -0.195 | 0.823(0.511,1.327) | 0.424 |
| age(3) | 0.953 | 2.593(0.766,8.784) | 0.126 |
| **BMI** |  |  | 0.000 |
| BMI(1) | 0.776 | 2.173(0.904,5.223) | 0.083 |
| BMI(2) | 1.359 | 3.893(1.561,9.708) | 0.004 |
| **DP** | 0.010 | 1.010(0.999,1.022) | 0.063 |
| **WBC** | 0.098 | 1.103(1.048,1.162) | 0.000 |
| **LYMPHp** | -0.028 | 0.973(0.951,0.994) | 0.013 |
| **HCT** | -0.085 | 0.919(0.895,0.943) | 0.000 |
| **MCV** | -0.049 | 0.953(0.928,0.977) | 0.000 |
| **MCHC** | 0.006 | 1.006(0.999,1.012) | 0.109 |
| **PLT** | -0.006 | 0.994(0.991,0.997) | 0.000 |
| **MPV** | 0.072 | 1.075(0.913,1.266) | 0.387 |
| **Training set** | 0.787 (0.757–0.817) | | |
| **Validation set** | 0.833 (0.793–0.874) | | |

* with the first of these categories serving as the reference.

**Table 4 Summary of 2*2 table of confusion matrix for training and validation datasets**

|  | **Actual Outcome** | |
| --- | --- | --- |
| **Predicted Outcome** | **HAPE** | **non-HAPE** |
| **Training data set** |  |  |
| HAPE | 279 | 115 |
| non-HAPE | 136 | 334 |
| **Validation data set** |  |  |
| HAPE | 142 | 54 |
| non-HAPE | 47 | 148 |
| **Whole data set** |  |  |
| HAPE | 421 | 156 |
| non-HAPE | 183 | 495 |

**Table 5 Summary of 2*2 table of confusion matrix for training and validation datasets**

|  | **Total** | | | **male** | | | **female** | | |
| --- | --- | --- | --- | --- | --- | --- | --- | --- | --- |
|  | **β Coefficient** | **OR** | **P** | **β Coefficient** | **OR** | **P** | **β Coefficient** | **OR** | **P** |
| **Intercept** | 6.482 | NA | 0.000 | 8.344 | NA | 0.000 | 5.638 | NA | 0.023 |
| **gender(1)** | 1.063 | 2.895 | 0.000 |  |  |  |  |  |  |
| **age** |  |  | 0.787 |  |  | 0.012 |  |  | 0.006 |
| age(1) | -0.309 | 0.734 | 0.54 | 0.982 | 2.669 | 0.119 | -2.274 | 0.103 | 0.016 |
| age(2) | -0.352 | 0.703 | 0.48 | 0.588 | 1.8 | 0.344 | -1.606 | 0.201 | 0.085 |
| age(3) | -0.452 | 0.637 | 0.376 | 0.207 | 1.229 | 0.745 | -1.2 | 0.301 | 0.204 |
| **BMI** |  |  | 0.000 |  |  | 0.000 |  |  | 0.014 |
| BMI(1) | -1.650 | 0.192 | 0.000 | -1.471 | 0.23 | 0.005 | -1.678 | 0.187 | 0.014 |
| BMI(2) | -0.694 | 0.499 | 0.000 | -0.638 | 0.529 | 0.000 | -0.672 | 0.51 | 0.02 |
| **WBC** | 0.112 | 1.119 | 0.000 | 0.103 | 1.109 | 0.000 | 0.115 | 1.122 | 0.004 |
| **LYMPHp** | -0.027 | 0.974 | 0.003 | -0.032 | 0.969 | 0.004 | -0.025 | 0.975 | 0.17 |
| **HCT** | -0.092 | 0.912 | 0.000 | -0.096 | 0.909 | 0.000 | -0.092 | 0.912 | 0.000 |
| **MCV** | -0.037 | 0.964 | 0.000 | -0.044 | 0.957 | 0.001 | -0.018 | 0.982 | 0.301 |
| **MCHC** | 0.005 | 1.005 | 0.088 | 0.005 | 1.005 | 0.262 | 0.004 | 1.004 | 0.279 |
| **PLT** | -0.007 | 0.994 | 0.000 | -0.007 | 0.993 | 0.000 | -0.007 | 0.993 | 0.000 |
| **MPV** | 0.015 | 1.015 | 0.592 | 0.013 | 1.013 | 0.725 | -0.004 | 0.996 | 0.915 |
| **DP** | 0.006 | 1.006 | 0.155 | 0.000 | 1.000 | 0.959 | 0.02 | 1.020 | 0.038 |
| **ROC** | 0.797(0.773,0.822) |  | 0 | 0.796(0.766,0.827) |  | 0 | 0.817(0.775,0.859 |  | 0 |

*The models were adjusted for age, gender, and BMI, with the first of these categories serving as the reference..
